# Organic Matter and Total Nitrogen Lead to Different Microbial Community Structure in Sediments Between Lagoon and Surrounding Areas by Regulating *Xenococcus* Abundance

**DOI:** 10.3389/fmicb.2022.859921

**Published:** 2022-04-21

**Authors:** Yonggan Chen, Minjing Zheng, Yue Qiu, Hong Wang, Haonan Zhang, Qiongren Tao, Hongwei Luo, Zhenhua Zhang

**Affiliations:** ^1^Key Laboratory of Utilization and Conservation for Tropical Marine Bioresources (Hainan Tropical Ocean University), Ministry of Education, Sanya, China; ^2^Hainan Key Laboratory for Conservation and Utilization of Tropical Marine Fishery Resources, Hainan Tropical Ocean University, Sanya, China; ^3^Key Laboratory of Biodiversity and Biosafety, Nanjing Institute of Environmental Sciences, Nanjing, China

**Keywords:** organic matter, total nitrogen, microbial community, sediments, lagoon, *Xenococcus*

## Abstract

Coastal lagoon is an important productive ecosystem on the Earth. In this study, we compared microbial community in the sediments between lagoon and surrounding areas, and explored mechanism for the variation of microbial community. As a result, the sediment of surrounding area showed significantly higher organic matter and total nitrogen than that of the lagoon. The linear regression analysis revealed that organic matter and total nitrogen are positively correlated with *Xenococcus*. Bacterial and fungal PCoA1 showed significantly positive relationships with the relative abundance of *Xenococcus*, indicating that *Xenococcus* affects the bacterial and fungal community in the sediments of both the lagoon and surrounding area. ANOSIM analysis demonstrated that there were significant differences in bacterial and fungal community structure in the sediments between the lagoon and surrounding areas. Therefore, organic matter and total nitrogen affect the microbial community structure in the sediments of lagoon and surrounding areas by regulating the abundance of *Xenococcus*.

## Introduction

Lagoon is a highly productive ecosystem that plays important ecological roles, such as biogeochemical element cycling, coastal biodiversity and blue carbon sequestration (Bhatnagar et al., 2020). The status of lagoon may vary in response to natural events and human activities (Duran et al., 2015). Microbial community shaped biogeochemical cycle, they drive carbon cycling in lagoon sediments, mediate mineral nutrition of sea plants, and alleviate carbon limitations of other sediment organisms. The activity of microbial community is crucial for carbon, nitrogen, and phosphorus cycling (Mohit et al., 2014), as well as determines the fate of nutrition (Bordenave et al., 2004a,b; Pringault et al., 2008; Stauffert et al., 2013; M’rassi et al., 2015).

More insights in the microbial community are critical for understanding the functions of lagoon ecosystem. For instance, Sáenz de Miera et al. (2021) reported that the seasonal disturbance of water level cycles is the major determinant of microbial community structure in sediments of saline lagoons located in the northwest of Spain. Ben Salem et al. (2019) revealed bacterial community assemblages in sediments under high anthropogenic pressure at Bizerte Lagoon hydrological system, Tunisia. Proteobacteria, Bacteroidetes, Chloroflexi, Planctomycetes, and Nitrospirae are the major phyla, which is consistent with previous observations in sediment environments (Behera et al., 2017; Fan et al., 2017). Besides the popularly abundant phyla Proteobacteria, Chloroflexi and Planctomycetes, Spirochaetes were also found in the sediment of an enclosed shallow coastal lagoon, Magdalen Islands, Gulf of St. Lawrence, Canada (Mohit et al., 2015).

As a matter of fact, coastal lagoons usually have strong physicochemical gradient of salinity, nutrient concentration, turbidity, and organic matter content. The diversity of microorganisms in these ecosystems indicates that microbial community can be shaped by a variety of environmental factors, such as salinity, temperature, pH, hydrology, and latitude (Mohit et al., 2014). A previous study has revealed the environmental factors controlling the bacterial abundance in the sediments of a Mediterranean lagoon ecosystem. In this study, the variation of bacterial distribution was related to abiotic (temperature, ammonium, and pH) and trophic parameters (such as chlorophyll *a*) (Pala et al., 2018). An association analysis between sediment bacterial communities and environmental factors in Intermittently Closed and Open Lakes and Lagoons (ICOLLs) revealed that the bacterial diversity in Manly may be due to eutrophication, and that in Narrabeen may be attributed to the variety of organic matter sources (Filippini et al., 2019). Another study investigated the bacterial diversity and microbial functional response to organic matter composition in deltaic lagoon sediments, finding that at low organic contamination levels, the riverine inputs provide additional bioavailable carbon sources without evident adverse effects (Zoppini et al., 2020).

Coastal lagoon and the surrounding area are important aquatic systems with strong physicochemical gradients, where microorganisms have been well recognized to participate in biogeochemical cycles (Mary et al., 2014). However, the microbial composition, distribution and function in these environments remain largely unknown in the South China Sea. In this study, we investigated the microbial community in Qilianyu lagoon of China by analyzing the microorganisms involved in the regulation of microbial community structure with high-throughput sequencing, and systematically studying the correlation between microorganisms and physicochemical characteristics.

## Materials and Methods

### Study Site and Sample Collection

Qilianyu lagoon is located at 16.9703–16.9830N 112.2293–112.2585E. Two areas were selected for analysis, including lagoon zone (IL) and surrounding area (OL). The samples were collected in August 2020. Ten different sites were selected in the lagoon and surrounding areas for the study, respectively. Quadruplicate 1 × 1 m^2^ plots were constructed in each site. For each plot, five soil cores (5–10 cm in diameter; 1–10 cm in depth) were taken to make a composite sample.

### Determination of Physicochemical Characteristics

Sediment samples were air-dried and sieved through a 100-mesh sieve for determination of organic matter (OM). pH, total nitrogen (TN), sodium (Na), nitrate (${\text{NO}}_{3}^{-}$-N) and ammonium (${\text{NH}}_{4}^{+}$-N) were determined after sieving through a 10-mesh sieve. Available potassium was determined using the method described by Easton and Lovering (1964). Other physicochemical characteristics were tested according to the method described by Chen et al. (2020).

### Quantification of Microbial Abundance

Genomic DNA was extracted from soil samples using the OMEGA Soil DNA Kit (M5635-02) (Omega Bio-Tek, Norcross, GA, United States). NanoDrop ND-1000 spectrophotometer (Thermo Fisher Scientific, Waltham, MA, United States) and agarose gel electrophoresis were used to measure the quantity and quality of DNA. Bacterial and fungal abundance was determined by real-time quantitative polymerase chain reaction (Chen et al., 2021). The primers 338F (5’-ACTCCTACGGGAGGCAGCA-3’) and 519R (5’-ATTACCGCGGCTGCTGG-3’) (Zhang et al., 2017) and the primers ITS5 (5’-GGAAGTAAAAGTCGTAACAAGG-3’) and ITS2 (5’-GCTGCGTTCTTCATCGATGC-3’) (White et al., 1990) were used to evaluate the 16S rRNA and ITS gene copy number, respectively.

### PCR Amplification and Sequencing

Primer 338F (5’-ACTCCTACGGGAGGCAGCA-3’) and primer 806R (5’-GGACTACHVGGGTWTCTAAT-3’) were used to amplify 16S rDNA V3–V4 region (Hong et al., 2020). Primers ITS5 (5’-GGAAGTAAAAGTCGTAACAAGG-3’) and ITS2 (5’-GCTGCGTTCTTCATCGATGC-3’) were used to amplify the ITS1 region (White et al., 1990). PCR amplification and product purification were carried out as described by Chen et al. (2021). Sequencing was performed using the Illumina MiSeq platform at Shanghai Personal Biotechnology Co., Ltd.

### Sequence Analysis

QIIME 2 was used to process the sequencing data (Bolyen et al., 2018). DADA2 plugin was then used for sequence quality filtering, denoising, merging and chimera removing (Callahan et al., 2016). Non-singleton amplicon sequence variants (ASVs) were aligned with MAFFT (Katoh et al., 2002) and used to construct phylogenetic trees with FastTree2 (Price et al., 2009). Bacterial and fungal taxonomy was assigned to ASVs using the classify-sklearn naïve Bayes taxonomy classifier in feature-classifier plugin (Bokulich et al., 2018) against the SILVA Release 132 /UNITE Release 8.0 database, respectively (Kõljalg et al., 2013).

### Bioinformatics and Statistical Analysis

QIIME2 and R packages (v3.2.0) were used to analyze the sequence data. The observed species, Chao1, Shannon, Simpson, Faith’s PD, Pielou’s evenness, Good’s coverage, and beta diversity metrics Bray-Curtis dissimilarity were estimated by the diversity plugin. The alpha-diversity was visualized as box plots, and the beta diversity was visualized via principal coordinate analysis (PCoA). Significant differences in microbial structure were evaluated based on ANOSIM (Analysis of similarities) (Clarke, 1993; Warton et al., 2012) performed by QIIME2. Venn diagrams were generated using the R package “VennDiagram” (Zaura et al., 2009). LEfSe (Linear discriminant analysis effect size) was performed to detect differentially abundant taxa across groups using the default parameters (Segata et al., 2011). Linear relationships analysis was performed by the genescloud tools, a free online platform for data analysis^1^. The environmental factors affecting microbial community was analyzed by Canonical Correlation Analysis (CCA) using the *vif.cca*, *step model*, and *anova* functions within the R package (Oksanen et al., 2016).

### Sequence Accession Numbers

The bacterial and fungal sequence data are deposited in China National Microbiology Data Center (NMDC) with accession numbers NMDC10017942 and NMDC10017943, respectively.

## Results

### Site Characteristics

The sampling sites were distributed in the lagoon and surrounding areas (Supplementary Figure 1). Physicochemical details of the selected sampling sites are presented in Table 1, which can reflect the ecosystem characteristics of the lagoon. No significant difference was observed in sodium, ${\text{NH}}_{4}^{+}$-N and ${\text{NO}}_{3}^{-}$-N between the samples from the two kinds of sites (*P* > 0.05). Furthermore, OL sample showed higher organic matter (0.46 ± 0.05%), total nitrogen (0.33 ± 0.05 g/kg), pH (8.12 ± 0.05) and available potassium (174.95 ± 15.76 mg/kg) than IL sample (*P* < 0.05). A detailed analysis of the data indicated that sediments in the lagoon and surrounding areas were nutrient-deficient and pollution-free.

**TABLE 1:** Physicochemical parameters measured for the sample sites in Qilianyu lagoon and surrounding areas.

Samples	pH	OM (%)	Total nitrogen (g/kg)	Available potassium (mg/kg)	sodium (mg/kg)	${\text{NH}}_{4}^{+}$-N (mg/kg)	$\text{N}\,O_{3}^{-}$-N (mg/kg)
OL	8.12 ± 0.05 a	0.46 ± 0.05 a	0.33 ± 0.05 a	174.95 ± 15.76 a	4081.02 ± 361.04 a	1.35 ± 2.06 a	1.56 ± 1.03 a
IL	7.88 ± 0.11 b	0.36 ± 0.05 b	0.23 ± 0.03 b	153.57 ± 13.07 b	4114.89 ± 355.52 a	0.94 ± 0.37 a	2.61 ± 3.22 a

*The referred months of sampling were in 2020. Values are presented as the mean ± standard deviation (n = 10). Data of ${\text{NH}}_{4}^{+}$-N and ${\text{NO}}_{3}^{-}$-N were subjected to Kruskal-Wallis H test, and other data were subjected to t test. Different letters denote a significant difference (P < 0.05).*

### α-Diversity and β-Diversity

**FIGURE 1:**
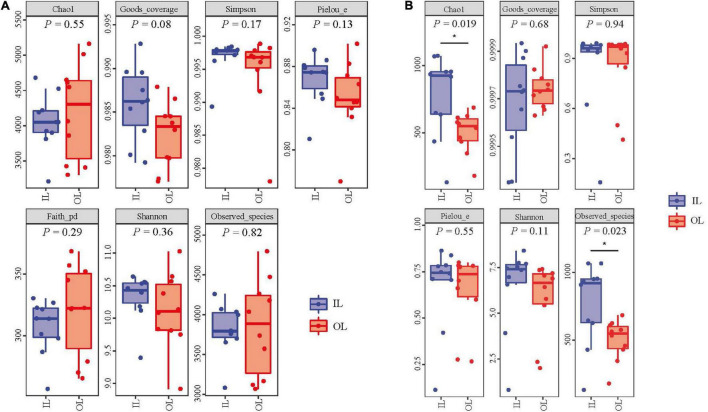
Bacterial **(A)** and fungal **(B)** α-diversity indices of the sediments from Qilianyu lagoon and surrounding area.

No significant differences were observed in fungal abundance of the two samples, whereas IL sample 3.55 × 10^9^ copies/g was higher than OL sample 1.94 × 10^9^ copies/g in bacterial abundance (*P* < 0.05). The rarefaction curves were smooth for both the OL and IL samples, indicating that the sequencing depth could well reflect the structural characteristics of the microorganisms collected from two different sites. In addition, Figure 1 shows that the Good’s coverage values of sediments all reached above 0.980, suggesting that the sequencing results can represent the real situation of microorganisms in the sediments of different sampling sites. Shannon, Simpson and Pielou’s evenness index of bacteria (including Faith’s PD) and fungi showed no difference in two kinds of samples. However, the Chao1 and Observed species index indicated that the total number of fungal species in the IL sample was larger than that in the OL sample (*P* < 0.05) (Figure 1B).

**FIGURE 2:**
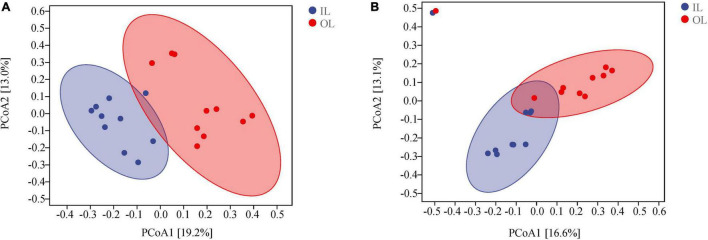
Microbial community structures in the sediments of Qilianyu lagoon and surrounding area. Bray-curtis principal coordinate analysis of bacterial **(A)** and fungal **(B)** community structures.

According to the PCoA analysis (Figures 2A,B), the first two principal components of bacteria account for 19.2 and 13.0% based on the Bray-curtis distances. For fungi, the first two principal components could account for 16.6 and 13.1%. According to the ANOSIM analysis, there were significant differences in bacterial and fungal structure between the OL and IL samples (*P* < 0.05). These results indicated that the microorganisms existing in sediments are significantly different in the lagoon and surrounding areas.

### Microbial Community Composition and Structure

**FIGURE 3:**
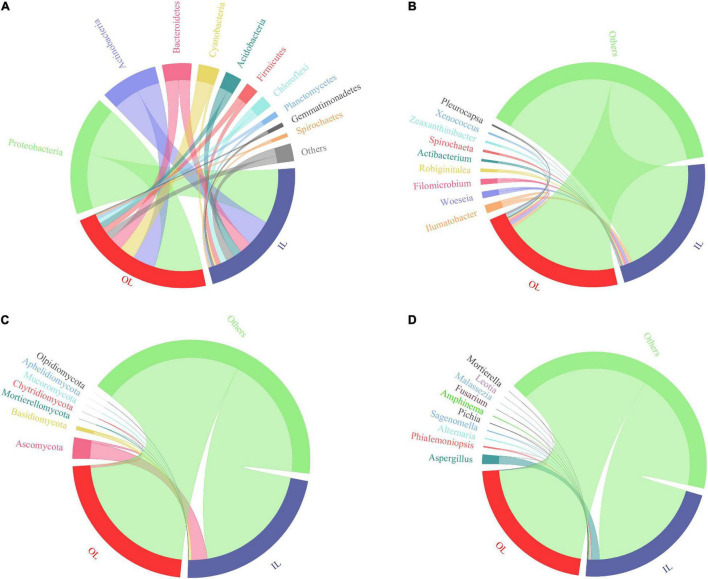
Relative abundance of microbial taxa in the sediments from Qilianyu lagoon and surrounding area. Bacterial phyla **(A)** and genera **(B)**; and fungal phyla **(C)** and genera **(D)**.

Figures 3A,B show the bacterial community composition at the phylum and genus levels. Proteobacteria, Actinobacteria, Bacteroidetes, Cyanobacteria and Acidobacteria were predominant in the sediments of the lagoon and surrounding areas. Compared with that in IL samples (1.8945 ± 1.3869%), the relative abundance of Cyanobacteria in OL samples was significantly increased to 12.0615 ± 9.7312% (*P* < 0.05). Typically, *Xenococcus* which belongs to Cyanobacteria was present in both the IL and OL samples, accounting for 0.1675 ± 0.1123% and 1.0191 ± 0.7042% of the bacterial community, respectively (*P* < 0.05). Besides, *Pleurocapsa* was also present in both the IL and OL samples, accounting for 0.0228 ± 0.0207% and 1.0605 ± 1.7925% of the bacterial community, respectively (*P* < 0.05).

Fungal ASVs were predominantly associated with the phyla of Ascomycota, Basidiomycota, Mortierellomycota, Chytridiomycota and Mucoromycota (Figure 3C). Basidiomycota was more abundant in the IL sample than in the OL sample (*P* < 0.05), and other phyla were similar in the IL and OL samples. *Phialemoniopsis* was relatively more abundant (*P* < 0.05) in the lagoon sediments (0.8639 ± 1.2013%) than in the surrounding sediments (0.0806 ± 0.2467%). Differently, *Aspergillus*, *Alternaria*, *Sagenomella*, *Pichia*, *Amphinema*, and *Fusarium* showed no obvious difference in the two kinds of samples (Figure 3D).

**FIGURE 4:**
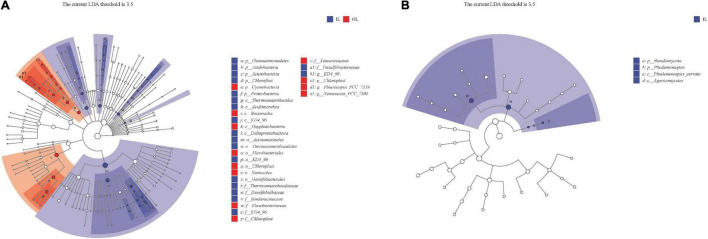
Clade diagram of the LDA scores computed for differentially abundant bacterial **(A)** and fungal **(B)** genera in the sediments between Qilianyu lagoon and the surrounding area.

LEfSe was used to further analyze bacterial and fungal genera in the sediments of the lagoon and surrounding areas. The threshold of 3.5 was employed on the logarithmic LDA score to determine the discriminative feature. Cyanobacteria and Bacteroidia were hyper-dominant in the sediments of the surrounding area, whereas Gemmatimonadetes, Acidobacteria, Actinobacteria, Chloroflexi and Proteobacteria were the most dominant phyla (accounting for 75.84% of the total bacterial phyla) in the sediments of the lagoon (Figure 4A). The bacterial genera *Xenococcus* and *Pleurocapsa* were more abundant in the sediments of the surrounding area. As for fungi, Basidiomycota and Phialemoniopsis were more abundant in the sediment of lagoon. However, there was no discrepancy in fungal genera in the two kinds of sediments (Figure 4B).

### Relationships of Bacterial Abundance With Physicochemical Characteristics and Microbial Indicators

**FIGURE 5:**
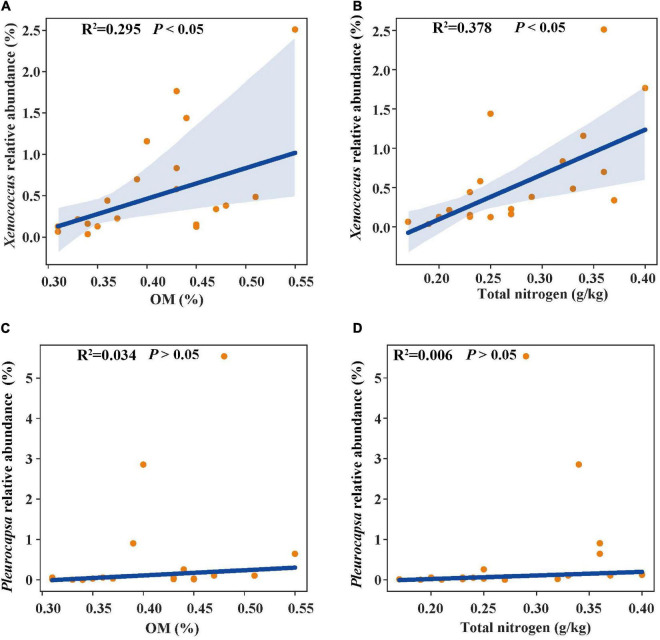
Linear regression relationship between organic matter **(A)**, total nitrogen **(B)** and *Xenococcus* relative abundance; organic matter **(C)**, total nitrogen **(D)** and *Pleurocapsa* relative abundance.

**FIGURE 6:**
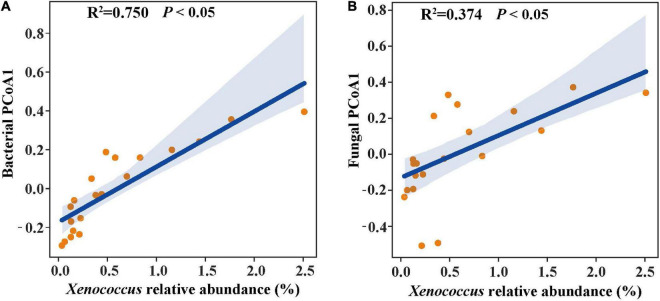
Linear regression relationship of the relative abundance of *Xenococcus* with bacterial structure **(A)** and fungal structure **(B)**.

**FIGURE 7:**
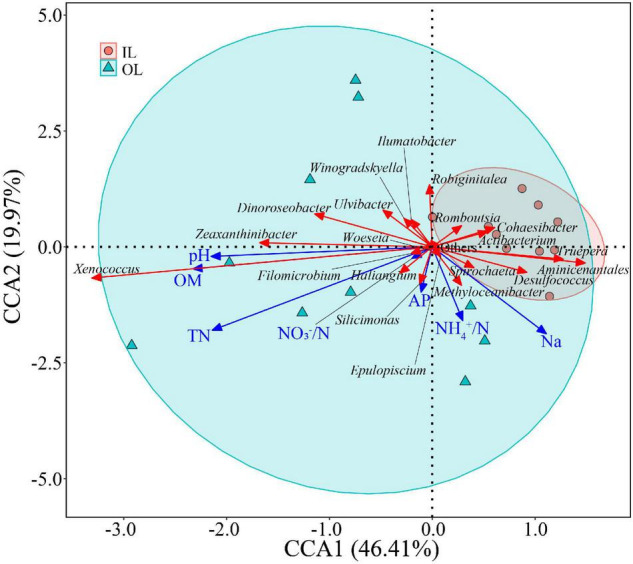
Relationships between sediment microbial communities and environmental factors indicated by Canonical Correlation Analysis (CCA). IL (red circle) and OL (blue triangle) represent sediments from lagoon zone and surrounding area, respectively.

In the linear regression analysis, organic matter and total nitrogen exhibited positive relationships with *Xenococcus* abundance (Figures 5A,B). However, a bacterial genus *Pleurocapsa* exhibited no linear relationship with organic matter and total nitrogen (Figures 5C,D). PCoA1 was selected as a microbial indicator in the linear model. As a result, the bacterial and fungal PCoA1 (*P* < 0.05) showed significant positive relationships with the relative abundance of *Xenococcus*. These results indicated that organic matter and total nitrogen could significantly regulate the relative abundance of *Xenococcus*, but have no correlation with *Pleurocapsa*. Furthermore, *Xenococcus* affected the bacterial and fungal community structure in the sediments of the lagoon and surrounding areas (Figures 6A,B). To identify the relationship between environmental variables and bacterial community, CCA was applied to interpret the pattern for sediment samples at the genus levels. As shown in Figure 7, 46.41% (*P* = 0.001 permutation test) and 19.97% (*P* = 0.005 permutation test) of the cumulative variance of the relationship were explained by the first and second axes of CCA, respectively, which indicated that the environmental variables could mostly explain (66.38%) the distribution of the genus in our study. In addition, organic matter, total nitrogen, and pH significantly affected (*P* < 0.05) *Xenococcus* which consistent with the above linear regression analysis. Furthermore, ${\text{NH}}_{4}^{+}$-N and ${\text{NO}}_{3}^{-}$-N contents significantly affected (*P* < 0.05) *Haliangium*, *Silicimonas* and *Methyloceanibacter*, indicating that above genus may related to the nitrification processes in lagoons sediments. The samples from OL and IL cannot be separated from CCA analysis. However, samples from IL clustered more together than OL, which indicated that the IL bacterial community structure remained relatively stable.

## Discussion

Proteobacteria are frequently reported as the dominant phyla in lagoon sediments, and microbial composition will change according to their geographical location (Behera et al., 2017). Zoppini et al. (2020) found that Proteobacteria and Bacteroidetes were dominant in the deltaic lagoon sediments of Italy, followed by Epsilonbacteraeota, Firmicutes, Chloroflexi, Planctomycetes and Verrucomicrobia. A previous study investigated the benthic sediment bacteria in Celestún Lagoon in the Southern Gulf of Mexico. The results revealed that Proteobacteria, Chloroflexi, Bacteroidetes and Planctomycetes are the dominant taxa, followed by Spirochaetae, Acidobacteria, Actinobacteria, Cyanobacteria and Verrucomicrobia (Cadena et al., 2019). Sáenz de Miera et al. (2021) collected sediments from four lagoons of Spain, including Barillos, Salina Grande, Villarrin and Villalpando. Proteobacteria was identified as the most abundant phylum in all lagoons, except for Grande, where Chloroflexi was more abundant than Proteobacteria despite insignificant differences. The popular marine bacteria Proteobacteria were also found to be dominant in the sediments of Qilianyu lagoon. However, the order of other bacterial phyla in Qilianyu lagoon was different from that in the aforementioned lagoons. For fungi, ITS1 sequences of sediments collected from a coastal lagoon connected as a subestuary to Waquoit Bay (Cape Cod, MA, United States) were identified to be associated with Ascomycete, Chytridiomycota, Sordariomycetes, Pleosporales and Agaricomycetes. Typically, the sediments were dominated by the ascomycete genus *Orbilia*, which is different from the case of Qilianyu lagoon where *Aspergillus* is the main genus (Ortega-Arbulú et al., 2019). These results indicate that the divergence of bacterial and fungal composition in the sediments is affected by the geographical location.

Previous studies have identified total organic carbon as one of the most important factors affecting the bacterial composition in different aquatic habitats (Lallias et al., 2015; Highton et al., 2016). The relative abundance of bacterial taxa increased with total organic carbon increasing. These bacteria include Candidatus Giovannonibacteria (GW2011_GWB1_47_6b), Candidate division CPR3 (GW2011_GWF2_35_18), Parcubacteria (group bacterium ADurb. Bin326), Deferribacteraceae, Chlorobiaceae and Bradyamonadaceae (Filippini et al., 2019). A study of Chilika Lagoon located along the east coast of India also revealed the environmental conditions affected the microbial difference. The bacterial taxa include α-Proteobacteria, γ-Proteobacteria, Sphingobacteria, Bacilli, Nitrospira and Acidobacteria_Gp10, which prefer the higher salinity regime in the lagoon. In contrast, β-Proteobacteria, Clostridia and Actinobacteria prefer the freshwater-brackish salinity regime. However, none of the sediment parameters (total organic carbon, total nitrogen, total phosphorus, and pH) measured in the Chilika Lagoon was significantly correlated with the bacterial community composition (Behera et al., 2017). Here, we found that organic matter and total nitrogen exhibited positive relationships with *Xenococcus*, whereas had no linear relationship with *Pleurocapsa*. Our results suggest that natural changes in organic and nitrogen enrichment in the sediments can regulate the *Xenococcus* in Qilianyu lagoon.

*Xenococcus* classified in the phyla Cyanobacteria, it is widely found in the ocean ecology, but their biogeography and functions in lagoon environments is poorly understood. A case study of the Golden Bay mangrove in China demonstrated that cyanobacterial assemblages were dominated by Synechococcus and Xenococcaceae, and there was a clear shift of dominance from Synechococcus to Xenococcaceae toward the sea (Zhu et al., 2018). Rigonato et al. (2013) revealed that most of cyanobacteria were uncultivated (unclassified) and only a few Xenococcus sequences were detected in the mangrove soils in south-east Brazil. However, *Xenococcus* species have been detected on the trunks, roots, and pneumatophores of several mangrove trees (including *Avicennia marina*) with morphology-based methods (Alvarenga et al., 2015). *Xenococcus* was also found in the intertidal zones of the Portuguese coast, and in cyanobacterial mats retrieved from the La Réunion lagoon (Brito et al., 2012; Echenique-Subiabre et al., 2015). These results indicate the ubiquity of *Xenococcus* in the marine environment. *Xenococcus* one of genus of Cyanobacteria, and many research showed that Cyanobacteria play an important role in balance of nutrients, primarily carbon and nitrogen of marine ecosystems (Al-Thukair et al., 2007; Charpy et al., 2012). In this research, we also found that organic matter and total nitrogen could significantly regulate the relative abundance of *Xenococcus*. Furthermore, *Xenococcus* was found to affect the bacterial and fungal community structure in the sediments. Therefore, changes in organic matter and total nitrogen may lead to the differences in microbial community structure of the sediments between the lagoon and surrounding areas through the regulation of *Xenococcus* abundance.

## Conclusion

This study analyzed the physicochemical characteristics and microorganisms in the lagoon and surrounding areas, which is beneficial to further understand the ecological characteristics of Qilianyu lagoon. The results indicate that organic matter and total nitrogen lead to the differences in microbial community structure in sediments between the lagoon and surrounding areas through the regulation of *Xenococcus* abundance. In the future, further research can be focused on whether fungi are involved in the regulation of the microbial community structure and the bacterial regulation mechanism.

## Data Availability Statement

The datasets presented in this study can be found in online repositories. The names of the repository/repositories and accession number(s) can be found in the article/Supplementary Material.

## Author Contributions

YC: software. YQ, HW, and HL: formal analysis. YC, MZ, YQ, HW, and QT: investigation. YC, MZ, and HZ: writing – original draft preparation. YC and ZZ: writing, review, editing, project administration, and funding acquisition. ZZ: supervision. All authors contributed to the article and approved the submitted version.

## Conflict of Interest

The authors declare that the research was conducted in the absence of any commercial or financial relationships that could be construed as a potential conflict of interest.

## Publisher’s Note

All claims expressed in this article are solely those of the authors and do not necessarily represent those of their affiliated organizations, or those of the publisher, the editors and the reviewers. Any product that may be evaluated in this article, or claim that may be made by its manufacturer, is not guaranteed or endorsed by the publisher.

## Footnotes

1
https://www.genescloud.cn

## Supplementary Material

Supplementary Figure 1 Geographic information system map of Qilianyu lagoon, China. IL (red curve) and OL (blue curve) represent sediments from lagoon zone and surrounding area, respectively.
