# Supplementary figures and images for: Organic Matter and Total Nitrogen Lead to Different Microbial Community Structure in Sediments Between Lagoon and Surrounding Areas by Regulating Xenococcus Abundance
Source: Front Microbiol. 2022 Apr 21;13:859921. doi: 10.3389/fmicb.2022.859921 (PMC9069056; doi:10.3389/fmicb.2022.859921)

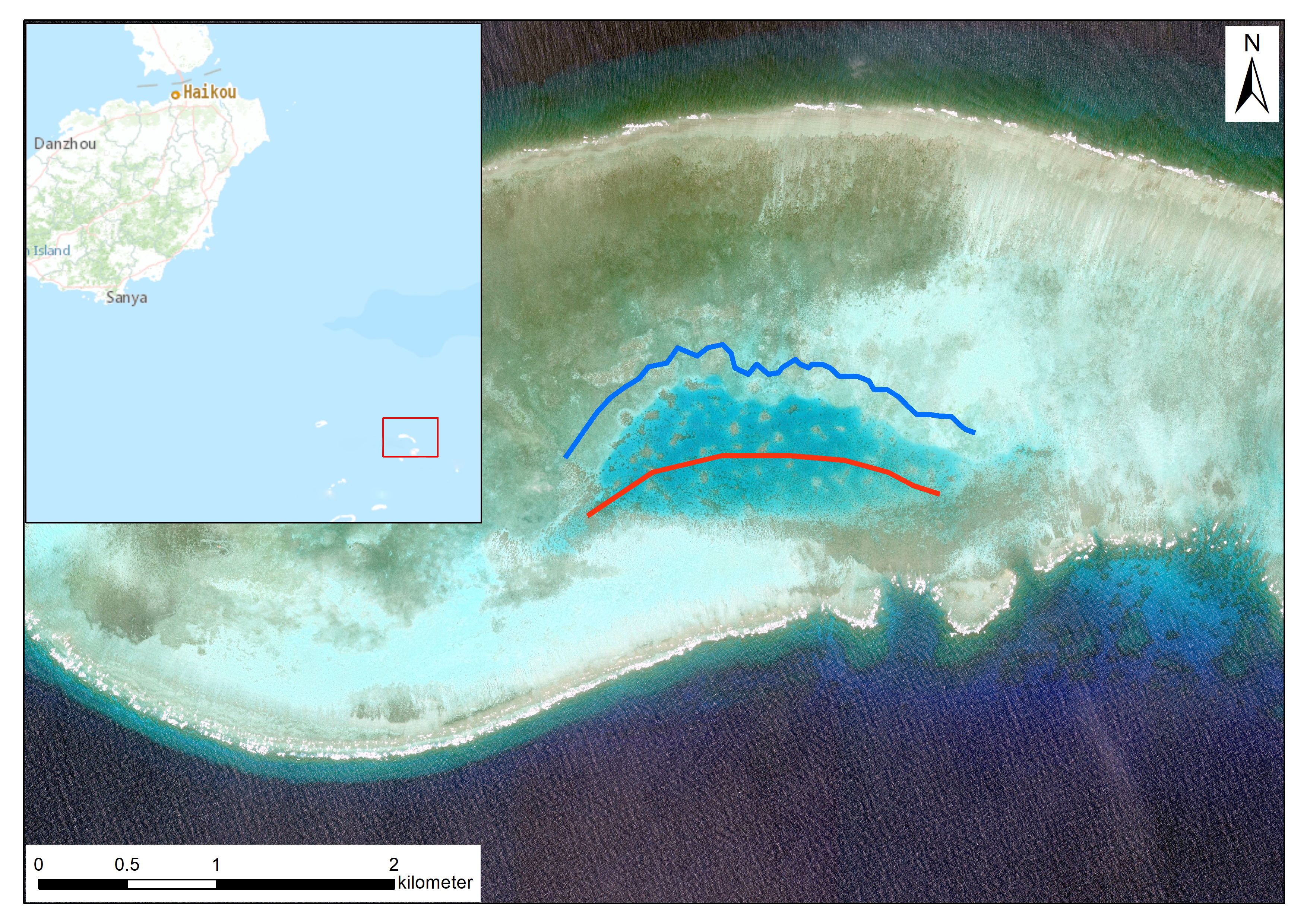

Supplement: Supplementary Figure 1 — Geographic information system map of Qilianyu lagoon, China. IL (red curve) and OL (blue curve) represent sediments from lagoon zone and surrounding area, respectively. [file Image_1.JPEG]
